# A Single Dose of a Psychedelic Drug Repairs Prefrontal Cortex Synaptic Physiology in a Mouse Model of Prenatal Alcohol Exposure

**DOI:** 10.1002/brb3.71406

**Published:** 2026-04-20

**Authors:** Tyler G. Ekins, Tao Deng, Omar J. Ahmed

**Affiliations:** ^1^ Department of Psychology University of Michigan Ann Arbor Michigan USA; ^2^ Michigan Psychedelic Center University of Michigan Ann Arbor Michigan USA; ^3^ Neuroscience Graduate Program University of Michigan Ann Arbor Michigan USA; ^4^ Department of Biomedical Engineering University of Michigan Ann Arbor Michigan USA; ^5^ Center for Computational Medicine & Bioinformatics University of Michigan Ann Arbor Michigan USA

**Keywords:** 25CN‐NBOH, intrinsic electrophysiology, patch‐clamp, prenatal alcohol, psychedelics, synaptic electrophysiology

## Abstract

**Background:**

Prenatal alcohol exposure (PAE) can cause fetal alcohol spectrum disorders (FASDs), which are characterized by neural circuit and behavioral dysfunction due to impaired brain development. At the neural and behavioral levels, PAE is associated with disrupted cortical synaptic transmission and lifelong impairments in learning and cognitive control. Despite the prevalence of FASDs (affecting up to one in 20 school‐aged children in the United States) and the associated personal, familial, and societal costs, there are currently no treatments to reverse neural circuit dysfunction.

**Methods:**

Using whole‐cell patch‐clamp electrophysiology, we investigated intrinsic excitability and synaptic activity in prefrontal cortex (PFC) pyramidal neurons from adolescent mice prenatally exposed to ethanol (6.6%) and later given a single injection of either saline or 25CN‐NBOH, a psychedelic neuroplastogen.

**Results:**

We found that PAE reduced intrinsic excitability and synaptic drive in PFC pyramidal neurons. 25CN‐NBOH treatment partially rescued intrinsic excitability deficits and restored synaptic drive.

**Conclusions:**

Psychedelic neuroplastogens may show promise as potential therapeutics for synaptic deficits associated with PAE and should be further explored in preclinical models.

## Graphical abstract

A single administration of the psychedelic drug 25CN‐NBOH repairs synaptic physiology and intrinsic excitability of prefrontal cortex layer 5 (L5) pyramidal cells in adolescent mice following prenatal alcohol exposure.

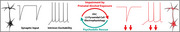

## 1 Introduction

Fetal alcohol spectrum disorders (FASDs) are lifelong conditions arising from prenatal alcohol exposure (PAE) and affect up to one in 20 school‐aged children (Centers for Disease Control and Prevention 2024). Fetal alcohol syndrome, the most severe presentation, occurs in about three per 10,000 live births and carries an average lifetime care cost approaching $2 million USD (Lupton et al. 2004). Given the high prevalence of FASDs and the burden on patients and families, there is an urgent need to develop therapeutic interventions.

Across clinical and preclinical studies, the prefrontal cortex (PFC), a hub for cognitive control, working memory, and flexible learning, has been identified as a key site of PAE‐related neural dysregulation (M. W. Miller 1986; Ferrer and Galofré 1987; E. K. Miller and Cohen 2001; Allan et al. 2014; Marquardt et al. 2014, 2020; Hamilton and Brigman 2015; Skorput et al. 2015; Skorput and Yeh 2016; Öztürk et al. 2017; Kable et al. 2020). Humans with FASDs have reduced density of dendritic spines, the postsynaptic sites of excitatory synapses, on PFC pyramidal neurons (Ferrer and Galofré 1987). Similarly, in rodent models of PAE, pyramidal neurons show fewer spines, and synaptic drive is altered in PFC (Reyes et al. 1983; Skorput et al. 2015; Skorput and Yeh 2016). Dysregulated PFC structure and neural activity are associated with behavioral impairments in executive function, learning, and cognitive control across PAE and other disorders (Liston et al. 2006; Astley et al. 2009; Minzenberg et al. 2009; Hart et al. 2013; Allan et al. 2014; Marquardt et al. 2014, 2020; Hamilton and Brigman 2015; Kaiser et al. 2015; Tang et al. 2019; Friedman and Robbins 2022). Yet, despite the high prevalence of FASDs, there are no known treatments that restore neural circuit function (Lupton et al. 2004; Wilhoit et al. 2017; Wozniak et al. 2019; Gomez and Abdul‐Rahman 2021; Popova et al. 2023).

Recent work suggests that serotonergic psychedelics may offer a mechanistically targeted path to neural circuit repair. A single psychedelic drug dose enhances synaptic connectivity in neocortical pyramidal neurons (Ly et al. 2018; Cameron et al. 2020, 2023; Revenga et al. 2021; Shao et al. 2021, 2025; Ekins et al. 2023, 2025; Jefferson et al. 2023; Vargas et al. 2023). These effects are mediated by serotonin 2A receptors (5‐HT_2A_Rs) (Ly et al. 2018; Cameron et al. 2020, 2023; Shao et al. 2021, 2025; Kwan et al. 2022; Vargas et al. 2023; Ekins et al. 2025). Importantly, 5‐HT_2A_R expression is unaltered by PAE (Tajuddin and Druse 1989; Kim et al. 1997), indicating that 5‐HT_2A_Rs remain relevant therapeutic targets for FASDs.

Given that PAE disrupts PFC synaptic connectivity, whereas psychedelic treatment enhances it, we hypothesized that psychedelic treatment in adolescent mice may correct PAE‐associated PFC circuit deficits. The selective 5‐HT_2A_R agonist 25CN‐NBOH (NBOH) was chosen as the psychedelic drug in this study because its acute actions on PFC layer 5 pyramidal neurons have been extensively characterized (Ekins et al. 2023; Wang et al. 2025), and single‐dose NBOH treatment produces a lasting elevation of synaptic connectivity in PFC neurons (Ekins et al. 2025) and a weeks‐long enhancement in reversal learning, a PFC‐dependent behavior (Brouns et al. 2025). We tested this hypothesis with whole‐cell patch‐clamp electrophysiology in adolescent mice and found psychedelic‐induced rescue of PFC synaptic activity and partial normalization of intrinsic excitability that was impaired by PAE. This work establishes a mechanistic framework for leveraging psychedelic‐driven plasticity to remediate PFC neural circuit pathology associated with PAE in a preclinical model.

## 2 Materials and Methods

### 2.1 Animals

All procedures and use of animals were approved by the University of Michigan Institutional Animal Care and Use Committee. Sample sizes were chosen based on previous publications (Ly et al. 2018; Nardou et al. 2019, 2023; Revenga et al. 2021; Shao et al. 2021; Ekins et al. 2023, 2025; Vargas et al. 2023). Eighty‐four total neurons from 19 (seven female, 12 male) adolescent mice, postnatal days 29–49 (P29–49), were used for this study. Experimental groups undergoing the PAE protocol and subsequent saline or psychedelic injections were all on a C57BL/6 background. Control data consisted of cell type‐, region‐, layer‐, and age‐matched neurons derived from control mice used in two of our recent studies (Ekins et al. 2023, 2025). These control data included Ai14, Ai32, Scnn1a‐Cre, Pvalb‐Cre, Grp‐Cre, Kj319‐Cre, and Htr2a‐flox, all on a C57BL/6 background. Notably, Htr2a‐flox mice were used only as controls (without Cre), as in our recent publication (Ekins et al. 2025). Additional details about these CRISPR‐Cas9‐generated Htr2a floxed mice are available in our recent publication (Ekins et al. 2025). All other lines were obtained from The Jackson Laboratory and/or bred in‐house and backcrossed to C57BL/6 mice. For additional comparisons with a limited subset of control data, genotypes were matched to those used in the PAE experiments (C57BL/6 and Pvalb‐Cre).

### 2.2 PAE

The PAE protocol used here was based on an established protocol using chronic alcohol administration to model heavy alcohol use during pregnancy, and prior studies using similar protocols have reported blood alcohol concentrations of approximately 80–150 mg/dL per day (Tajuddin and Druse 1989; Kim et al. 1997; Sickmann et al. 2014; Skorput et al. 2015, 2019; Skorput and Yeh 2016). Throughout pregnancy, drinking water was replaced with a 6.6% ethanol solution (Tajuddin and Druse 1989; Kim et al. 1997). At birth, the ethanol solution was removed, and standard drinking water was returned to the cage. Experimentation began in adolescence (P29–49).

### 2.3 Drugs

25CN‐NBOH, a selective 5‐HT_2A_R agonist (Fantegrossi et al. 2015; Buchborn et al. 2018, 2020; Märcher‐Rørsted et al. 2021; Odland et al. 2021; Wang et al. 2025), was purchased from Tocris Bioscience and was dissolved in sterile water before mixing with sterile saline for in vivo injections. Light sonication was used to assist solubilization in water. DMSO was avoided as a solvent as it is known to alter intrinsic electrophysiological properties of neocortical and hippocampal neurons at doses far lower than those regularly used in neuroscience experiments (Tamagnini et al. 2014). 25CN‐NBOH was administered via intraperitoneal injection at 10 mg/kg, selected based on our previous study, which found a long‐lasting enhancement in reversal learning after this dose (Brouns et al. 2025). After 24–48 h, electrophysiology experiments were conducted. The control group for this study was a region‐matched and age‐matched subset of control neurons from two of our recent datasets—one published (Ekins et al. 2025) and one preprinted (Ekins et al. 2023).

### 2.4 Slice Preparation

Mice were deeply anesthetized with isoflurane before decapitation. Brains were dissected out in ice‐cold sucrose‐substituted ACSF, saturated with 95% O_2_ and 5% CO_2_, and containing the following (in mM): 3 KCl, 1.25 NaH_2_PO_4_, 26 NaHCO_3_, 10 dextrose, 234 sucrose, 0.2 CaCl_2_, and 4 MgSO_4_. Coronal slices (300 µm thick) were prepared using a VT1200 vibratome (Leica) and placed in a high‐magnesium ACSF solution at 32°C for 30 min, after which the slices rested at room temperature for at least 30 more minutes prior to recording. During experiments, slices were incubated in a recording chamber with physiological temperature (32°C) ACSF (126 mM NaCl, 1.25 mM NaH_2_PO_4_, 26 mM NaHCO_3_, 3 mM KCl, 10 mM dextrose, 1.20 mM CaCl_2_, and 1 mM MgSO_4_) perfused at a rate of 3 mL/min. Slices containing the anterior cingulate cortex were obtained at anteroposterior coordinates relative to the bregma from 2.1 to −0.5 mm.

### 2.5 Layer 5 Regular Spiking Pyramidal Cell Recording and Quality Control

Neurons were visualized on an Olympus BX51WI microscope with an Olympus 60x water immersion lens and Andor Neo sCMOS camera (Oxford Instruments, Abingdon, Oxfordshire, UK). Patch electrodes were pulled from borosilicate glass (Sutter Instruments) and had diameters of 2–4 µm and resistances of 3–5 MΩ. Internal solution was potassium gluconate based and contained (in mM): 130 K‐gluconate, 0.6 EGTA, 10 HEPES, 2 Na_2_ATP, 0.3 NaGTP, 6 KCl, 3 MgCl_2_, and 0.5% biocytin (calculated chloride reversal potential of −68 mV; pH of 7.25; osmolarity of 290 mOsm). Pipette capacitance compensation and bridge balance were applied; recordings were not corrected post hoc for liquid junction potential. When conducting intracellular recordings in brain slice electrophysiology experiments, not all neurons in the brain slice are healthy or viable, which requires extensive quality control (Gouwens et al. 2019, 2020; Scala et al. 2019; Vormstein‐Schneider et al. 2020). To ensure high‐quality intracellular recordings, the following standard quality control metrics were used (Tricoire et al. 2011; Brennan et al. 2020, 2021; D'Amour et al. 2020; Ekins et al. 2020, 2023, 2025; Pelkey et al. 2020; Shao et al. 2021; Jedrasiak‐Cape et al. 2025): cells were excluded if baseline resting membrane potential was more depolarized than −50 mV or if uncompensated series resistance (*R*_s_) was >35 MΩ. All whole‐cell recordings were conducted with a MultiClamp 700B amplifier and digitized at 20 kHz with a Digidata 1550B (Molecular Devices) for acquisition on a computer equipped with pClamp 10.7 software (Molecular Devices). We included only putative intratelencephalic‐type pyramidal cells in the study, as identified by co‐expression of a regular spiking firing pattern and a physiologic index less than 5 (Baker et al. 2018; Gulledge 2024). Biocytin enabled post hoc streptavidin labeling and confocal imaging to confirm pyramidal cell morphology.

### 2.6 Current‐Clamp Recordings and Intrinsic Property Analysis

Current‐clamp experiments were conducted similarly to our previous studies (Cruikshank et al. 2012; Brennan et al. 2020, 2021; D'Amour et al. 2020; Ekins et al. 2020, 2023, 2025; Pelkey et al. 2020; Jedrasiak‐Cape et al. 2025). Membrane potentials were biased to −65 mV at the start of each sweep. Firing patterns were investigated using a series of 1‐s‐long current injections (step size 50 pA) up to 400 pA or until depolarization block was induced. Firing frequency was the number of spikes that occurred for each 1‐s‐long current step. Spikes that did not have a peak voltage reaching at least −10 mV were not counted. Frequency–current (*f*–*I* gain) was defined as the slope of a linear regression on firing frequency for each current injection from 0 pA to the injection at which maximum firing frequency was attained, or 400 pA, whichever occurred first. Total spike output was the sum of all spikes until maximum firing frequency or 400 pA. We analyzed intrinsic cell properties using Python (version 3.10.4) code with pyABF, NumPy, pandas, and Matplotlib as dependencies, as previously described (Ekins et al. 2023, 2025). For spike properties, we defined threshold as the voltage where the rise slope (*dV*/*dt*) reached 10 mV/ms. Amplitude was the difference between the threshold and the peak voltage. Half‐width was measured at the voltage corresponding to half‐maximal amplitude. Maximum rise slope was the maximum of *dV*/*dt*; maximum decay slope was the minimum of *dV*/*dt*. These properties were measured for the first spike evoked by the 1‐s current injection. Rheobase and latency to first spike were determined with a series of 2‐s‐long depolarizing current steps that were sufficient to evoke spiking (5–10 pA step size), repeated one to three times. Latency to first spike was determined by measuring the interval between the onset of the current step and the time at which the initial spike threshold was reached; rheobase was defined as the minimal current necessary to induce a spike during the 2‐s current step. Input resistance (*R*_in_), input capacitance (*C*_in_), and the membrane time constant (TC) were assessed with a series of small hyperpolarizing current steps (typically −10 to −20 pA, 20–40 sweeps). *R*_in_ was computed using the voltage difference between the maximum response during the current injection and the mean voltage during the 100 ms prior to the current injection, divided by the injected current. TC was computed by fitting an exponential decay curve to the voltage trace from the start of the current injection to the peak voltage deflection. Input (membrane) capacitance (*C*_in_) was calculated using the relationship *C*_in_ = TC/*R*_in_. Injection of hyperpolarizing current can produce a noticeable “sag,” which indicates the presence of a hyperpolarization‐activated cation current (*I*_h_), which activates after the initial hyperpolarization peak. Sag ratio was computed as the maximum response amplitude divided by the mean response amplitude of the last 50 ms of the current injection. Response amplitudes were computed relative to the baseline voltage, defined as the mean of the 100 ms prior to the current injection. Physiology index was computed following previously described methods (Baker et al. 2018; Gulledge 2024).

### 2.7 Voltage‐Clamp Recordings and Spontaneous Excitatory Postsynaptic Current (sEPSC) Analysis

Voltage‐clamp recordings were conducted at a holding potential of −70 mV, which yields approximately no driving force for chloride due to the internal solution composition (listed above). sEPSC recordings were taken in 30‐s sweeps with a brief hyperpolarizing test pulse (−5 mV, 250 ms) at the start to monitor *R*_s_ and *R*_in_ throughout the experiment. sEPSCs were detected using Easy Electrophysiology (version 2.6.1). sEPSCs were analyzed by an analyst blinded to experimental conditions in 30‐s sweeps (two to three analyzed per cell); the first second of each sweep was discarded because it contained the hyperpolarizing test pulse. Events were first identified with threshold‐based detection with the following settings: negative peak direction, 5‐ms local maximum period, 30‐ms decay search period, 8 pA threshold, 10‐ms search period, 1‐ms averaged baseline, and curved baseline and threshold. To eliminate false positives, events were manually inspected, and noise events were rejected. Frequency was determined for each sweep by dividing the total number of events per sweep by 29 s; amplitude was computed by averaging event amplitudes within each sweep; decay TC was determined by fitting an exponential to the averaged event for each sweep.

### 2.8 Cell Filling and Processing

To reveal cell morphology, biocytin (0.5%; Sigma–Aldrich) was added to the intracellular solution as in previous studies (Brennan et al. 2020, 2021; D'Amour et al. 2020; Ekins et al. 2020, 2023, 2025; Pelkey et al. 2020; Jedrasiak‐Cape et al. 2025). Biocytin slowly diffused into cells during the course of the recording (∼10 min). At the end of each recording, the patch pipette was slowly retracted from the cell to allow membrane resealing, then the slice was transferred to 4% PFA for overnight fixation. Afterward, each slice was washed in PBS and incubated for 24–48 h in streptavidin‐conjugated Alexa Fluor (488 or 647; Thermo Fisher Scientific) with 0.4% Triton X‐100 (Sigma–Aldrich). The fluorescent Nissl stain, NeuroTrace (435/455; Thermo Fisher Scientific), was added to the Alexa Fluor incubation step (at 1:200 dilution). After incubation, slices were washed in PBS, mounted on slides, and cover‐slipped using Fluoromount‐G Slides (Southern Biotech).

### 2.9 Statistics

Statistical analyses were performed in GraphPad Prism (version 10.5.0). Additional details on statistical tests are provided throughout the figure legends and Table S1.

## 3 Results

### 3.1 PAE Reduces Intrinsic Excitability and Synaptic Drive in PFC

**FIGURE 1:**
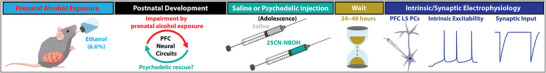
Experimental outline. Pregnant mice were given a solution of ethanol (6.6% volume/volume; v/v) in place of water until pups were born. After postnatal development, adolescent PAE mice (p30–45) were given a single injection of saline or the psychedelic drug 25CN‐NBOH (NBOH).

Adolescent PAE mice (Zhang 2004; Virtanen et al. 2018; Yang et al. 2018; Chen et al. 2025) received a single intraperitoneal injection of either saline or the psychedelic drug 25CN‐NBOH (NBOH; 10 mg/kg). At 24–48 h post injection, brain slices containing the PFC were prepared, and recordings were obtained from layer 5 (L5) pyramidal cells using whole‐cell patch‐clamp electrophysiology. Intrinsic excitability (membrane and firing properties) and synaptic input (sEPSCs) were assessed (Figure 1). Neuronal morphological reconstructions were done for representative neurons of each group, confirming the pyramidal morphology of recorded neurons (Figure S1).

**FIGURE 2:**
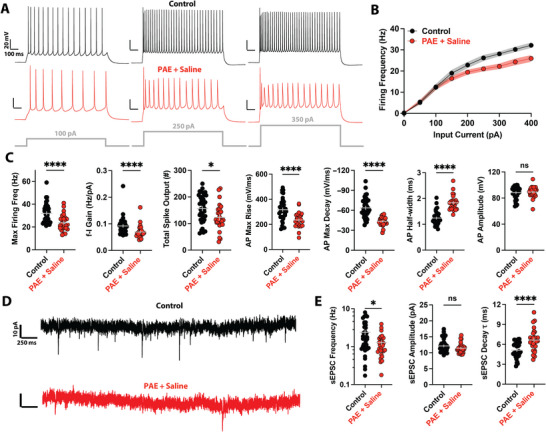
PAE reduces intrinsic excitability and synaptic input. (A) Voltage traces showing firing in response to different levels of current injections in single PFC L5 pyramidal neurons from control (top) and PAE + saline‐treated mice (bottom). Note the reduced firing, especially at the higher current injections. (B) *f*–*I* (firing frequency at each current injection value) curves show decreased spiking after PAE. (C) PAE reduces intrinsic excitability, altering many active membrane properties. (D) Current traces showing spontaneous excitatory postsynaptic currents (sEPSCs) in PFC L5 pyramidal neurons from control (top) and PAE + saline‐treated mice (bottom). Note the reduced number of synaptic currents (downward deflections) in the PAE trace. (E) PAE reduces the frequency and elongates the decay time constant of sEPSCs. *p < 0.05; ****p < 0.0001; ns, not significant. Error bars and shaded regions represent the standard error of the mean (SEM). Full statistical details are provided in Table S1.

First, we compared intrinsic excitability and synaptic properties in L5 pyramidal cells from PAE mice (treated only with saline) with those in control neurons from age‐matched mice. Relative to control neurons, PAE markedly decreased the intrinsic excitability of PFC neurons (Figure 2A). Frequency–current curves constructed from depolarizing step pulses revealed that PAE lowered *f*–*I* gain and reduced maximum firing frequency compared to control cells (Figure 2B,C). The total spike output of these neurons was also significantly lower (Figure 2C). Observed spike kinetics were consistent with alterations to voltage‐gated channels mediating spike generation: action potential maximum rise and fall slopes were reduced, and spikes were broader (Figure 2C). No changes were observed in spike amplitudes (Figure 2C), and the passive membrane properties did not differ significantly between groups (Figure S2).

Next, we examined the effects of PAE (again, only PAE mice treated with saline) on synaptic activity in PFC L5 pyramidal cells (Figure 2D). We found that PAE decreased the frequency of sEPSCs (Figure 2E), consistent with previous findings of reduced dendritic spine density and indicative of decreased synaptic connectivity (Reyes et al. 1983; Ferrer and Galofré 1987). The sEPSC amplitudes were unchanged, but kinetics were altered, as reflected by the longer sEPSC decay TCs in neurons from PAE mice (Figure 2E).

### 3.2 NBOH Treatment Boosts Neuronal Excitability and Synaptic Activity After PAE

**FIGURE 3:**
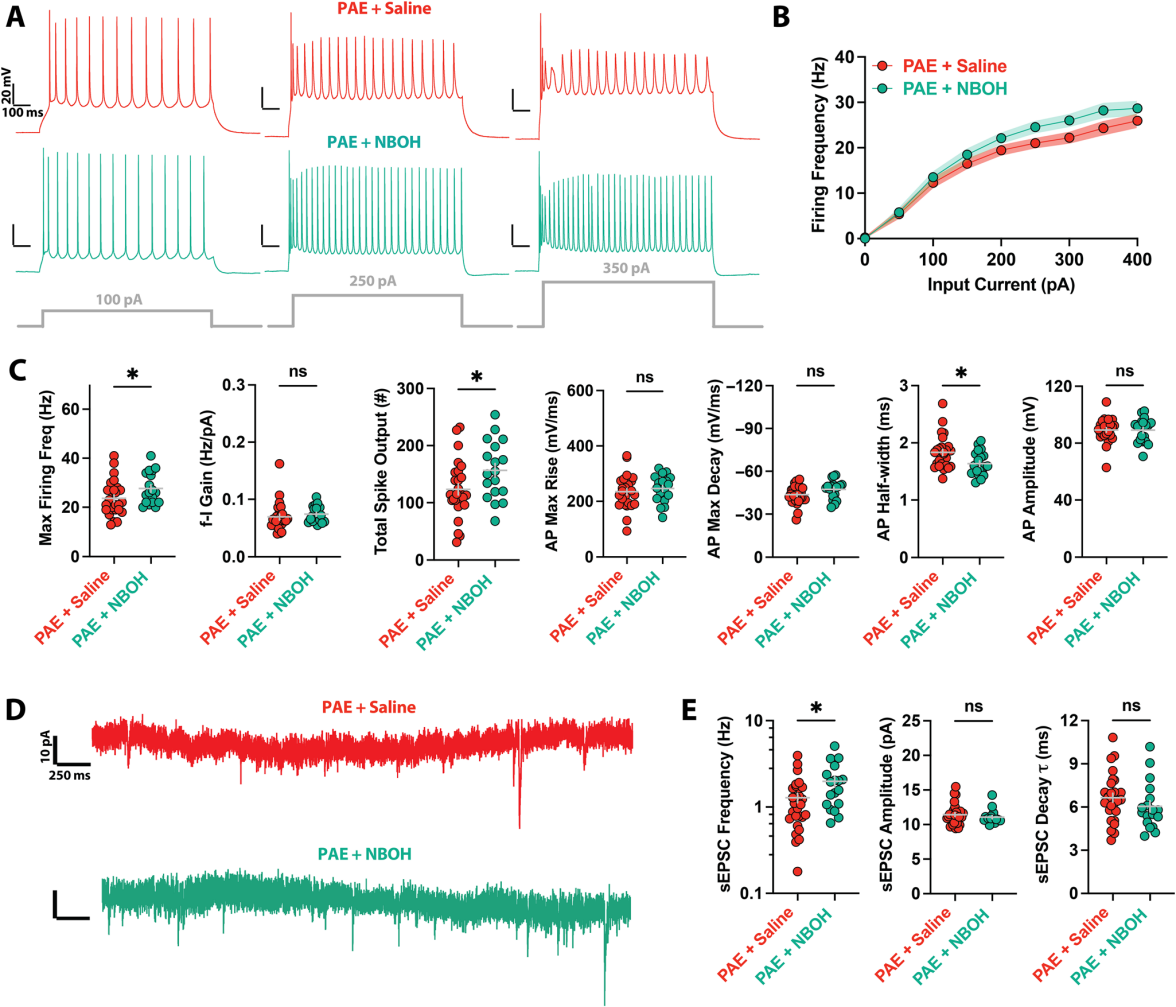
Psychedelic treatment boosts intrinsic excitability and synaptic input in neurons from PAE mice. (A) Voltage traces showing firing in response to different levels of current injections in single PFC L5 pyramidal neurons from PAE + saline‐treated mice (*top*) or PAE + psychedelic (NBOH)‐treated mice (*bottom*). Note the increased firing in the NBOH‐treated mice with the higher current injections. (B) *f*–*I* curves show increased spiking in neurons from PAE mice after NBOH treatment compared to saline treatment. (C) NBOH treatment enhances intrinsic excitability, altering several active membrane properties in PAE mice compared to saline treatment. (D) Current traces showing sEPSCs in PFC L5 pyramidal neurons from PAE + saline‐treated mice (*top*) or PAE + psychedelic (NBOH)‐treated mice (*bottom*). Note the increased number of synaptic currents in the NBOH trace. (E) NBOH treatment increases sEPSC frequency in neurons of PAE mice compared to saline treatment. *p < 0.05; ns, not significant. Error bars and shaded regions represent SEM. Full statistical details are provided in Table S1.

We next asked if treatment with a psychedelic drug (25CN‐NBOH; NBOH) would affect the electrophysiological properties of PFC L5 pyramidal cells compared to saline treatment in adolescent PAE mice. We found that a single dose of NBOH enhanced the intrinsic excitability of PFC L5 pyramidal cells relative to PAE with saline treatment (Figure 3A,B), significantly increasing maximum firing rates and total spike output compared to saline treatment (Figure 3C). While the *f*–*I* gain showed a trend toward an increase, this was not significant (Figure 3C). This result is comparable to previous studies’ findings that psychedelic treatment is without lasting impact on the intrinsic excitability of neocortical neurons from healthy mice (Cameron et al. 2023; Ekins et al. 2023, 2025) but partially normalizes intrinsic excitability alterations of neurons from chronically stressed mice (Lu et al. 2021). Spike waveform changes were selective: action potential half‐width was reduced, but spike rise and fall slopes were not significantly changed (Figure 3C). Again, no changes were observed in spike amplitudes (Figure 3C) or passive membrane properties (Figure S3). NBOH treatment of PAE mice significantly increased excitatory synaptic input (Figure 3D), boosting the sEPSC frequency compared to PAE mice given only a saline injection (Figure 3E). No significant changes were observed in either sEPSC amplitude or decay TC (Figure 3E).

### 3.3 NBOH Treatment Partially Normalizes PAE‐Associated PFC Neural Circuit Changes

**FIGURE 4:**
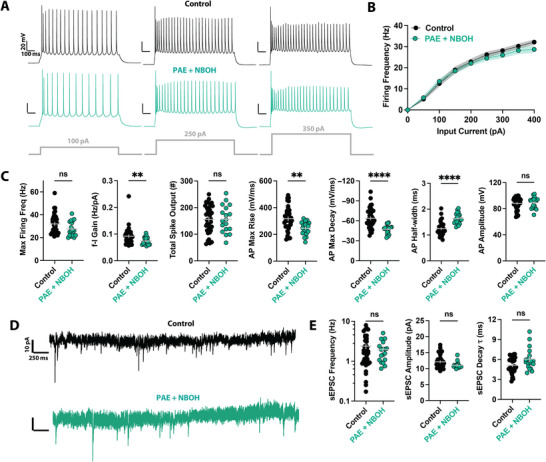
Psychedelic treatment partially normalizes PAE‐mediated intrinsic excitability deficits and restores synaptic input in neurons from PAE mice. (A) Voltage traces showing firing in response to different levels of current injections in single PFC L5 pyramidal neurons from control (*top*) or PAE + psychedelic (NBOH)‐treated mice (*bottom*). (B) *f*–*I* curves show that NBOH treatment partially corrects spiking in neurons after PAE. (C) NBOH treatment partially normalizes intrinsic excitability, reversing several active membrane property deficits associated with PAE. (D) Current traces showing sEPSCs in PFC L5 pyramidal neurons from control (*top*) or PAE + psychedelic (NBOH)‐treated mice (*bottom*). (E) NBOH treatment normalizes spontaneous excitatory synaptic deficits associated with PAE, restoring sEPSC frequency and decay time constant to control levels. **p < 0.01; ****p < 0.0001; ns, not significant. Error bars and shaded regions represent the standard error of the mean (SEM). Full statistical details are provided in Table S1.

Finally, to assess whether NBOH treatment corrects PAE‐induced synaptic input and intrinsic excitability deficits, we compared neurons from NBOH‐treated PAE mice to control neurons (Figure 4A,B). We found that while NBOH treatment increased intrinsic excitability compared to saline treatment in neurons from PAE mice, intrinsic excitability was only partially rescued compared to control neurons (Figure 4C). Maximum firing frequency and total spike output were not different from control neurons, but *f*–*I* gain was still lower (Figure 4C). Spike kinetics also showed residual abnormalities, with slower spike rise and fall slopes and broader half‐widths (Figure 4C). No changes were observed in spike amplitudes (Figure 4C) or passive membrane properties (Figure S4). In contrast, NBOH treatment restored excitatory synaptic input, as evidenced by the lack of statistically significant differences in sEPSC frequency, amplitude, or decay TC compared to control neurons (Figure 4D,E), and this pattern was unchanged in parallel analysis (Figure S5). Thus, NBOH treatment partially normalized intrinsic excitability deficits from PAE and restored excitatory synaptic drive to baseline levels (Table S1).

## 4 Discussion

Psychedelic medicine has shown great promise for treating neuropsychiatric disorders (Johnson et al. 2014, 2017; Bogenschutz et al. 2015; R. L. Carhart‐Harris et al. 2016, 2017, 2018; R. Carhart‐Harris et al. 2021; Sanches et al. 2016; Daws et al. 2022; Goodwin et al. 2022; Zaretsky et al. 2024). The neurobiological basis for psychedelic medicine is thought to involve long‐lasting enhancement of neuroplasticity and restoration of synaptic function (Revenga et al. 2021; Shao et al. 2021, 2025; Kwan et al. 2022; McClure‐Begley and Roth 2022; Ekins et al. 2023, 2025; Jefferson et al. 2023). Fetal alcohol spectrum disorders are also associated with decreased dendritic spine density and synaptic function in the PFC, and no current treatments exist to restore neural circuit function associated with PAE (Reyes et al. 1983; Ferrer and Galofré 1987; Wilhoit et al. 2017; Wozniak et al. 2019; Gomez and Abdul‐Rahman 2021; Popova et al. 2023).

PAE has been shown to disrupt PFC neuronal electrophysiology through changes beginning with dysfunctional neuronal migration, leading to neural circuit malformation and excitatory–inhibitory balance alterations (Cuzon et al. 2008; Giorgio and Granato 2015; Skorput et al. 2015; Skorput and Yeh 2016; Louth et al. 2018; Delatour et al. 2020). Dysfunction of glutamate receptors is also associated with PAE (Alhowail 2022). Early intervention has been attempted and shown success in treating some aspects of neural circuit dysfunction (Skorput et al. 2019). However, results here indicate that the psychedelic 25CN‐NBOH might be used to partially normalize alcohol‐induced changes to PFC circuits in adolescent mice in a preclinical model of PAE.

Our primary question was whether a single dose of NBOH could enhance synaptic input or increase intrinsic excitability compared to a single dose of saline in PFC L5 pyramidal cells from PAE mice (Figure 1). We found that overall, NBOH treatment significantly increased both sEPSC frequency and spiking ability of PFC L5 pyramidal cells (Figure 3). Saline‐treated PAE neurons had reduced intrinsic excitability and excitatory synaptic input related to a (cell type‐, region‐, layer‐, and age‐) matched dataset collected from healthy mice (Figure 2). However, neurons from NBOH‐treated PAE mice had similar levels of excitatory synaptic drive and fewer statistically insignificant differences in intrinsic excitability relative to healthy controls (Figure 4).

Psychedelic‐induced neuroplasticity depends on 5‐HT_2A_R activation (Ly et al. 2018; Revenga et al. 2021; Shao et al. 2021, 2025; Cameron et al. 2023; Ekins et al. 2023, 2025; Vargas et al. 2023), and 5‐HT_2A_R expression has been shown to be unaltered by PAE (Tajuddin and Druse 1989; Kim et al. 1997). Therefore, psychedelic neuroplasticity in PAE is expected to depend on activation of 5‐HT_2A_Rs. This should be examined in future studies. Furthermore, additional dosing paradigms, including different psychedelic drugs, dose levels, or repeated doses, should be explored. The utility of non‐psychedelic analogs, which have also been shown to induce neuroplasticity through activation of 5‐HT_2A_Rs (Cameron et al. 2020; Lu et al. 2021; Lewis et al. 2023; Qu et al. 2023; Aarrestad et al. 2025), should be explored for treating preclinical PAE.

Although this study provides evidence of partial normalization of synaptic and intrinsic electrophysiological properties in the PFC following psychedelic treatment in adolescent mice following PAE, this study should be interpreted with caution, as further studies are required to determine the effectiveness of psychedelic treatment in preclinical models for PAE. A single psychedelic dose has been shown to enhance neuroplasticity and improve cognitive flexibility for weeks to months (Shao et al. 2021, 2025; Jefferson et al. 2023; Fisher et al. 2024; Brouns et al. 2025). Future studies will determine if psychedelic neuroplastogens can induce a weeks‐ to months‐long neuroplasticity enhancement following PAE, or if the effects are short‐lived, and whether this differs in male and female mice. Both sex‐dependent (Shao et al. 2021; Jaster et al. 2022, 2025) and sex‐independent effects of psychedelic drugs have been found in prior preclinical studies (Ekins et al. 2023, 2025; Brouns et al. 2025; Shao et al. 2025). Furthermore, it is important to resolve the impact of psychedelic treatment on behavior in PAE mice. As PAE is associated with decreased cognitive flexibility (Alhowail 2022), it is essential to determine if psychedelic treatment can improve reversal learning in mice after PAE. The outcomes of these and other preclinical studies will better inform whether psychedelic neuroplastogens have promise in treating PAE‐induced neurobehavioral pathology.

## 5 Conclusion

These results imply that psychedelic (25CN‐NBOH) treatment may help normalize intrinsic and synaptic electrophysiological deficits in a mouse model of PAE. Of particular interest for treating FASDs is the use of non‐hallucinogenic analogs of psychedelic drugs. Both serotonergic psychedelic drugs and non‐hallucinogenic analogs induce neuroplasticity through activation of 5‐HT_2A_Rs and are often referred to as neuroplastogens (Cameron et al. 2020; Lu et al. 2021; Lewis et al. 2023; Qu et al. 2023; Aarrestad et al. 2025). In addition to increasing dendritic spine density, non‐hallucinogenic neuroplastogens also show promise in treating PFC‐dependent behavioral deficits across multiple preclinical models of neuropsychiatric disorders (Cameron et al. 2020; Lu et al. 2021; Lewis et al. 2023; Qu et al. 2023; Aarrestad et al. 2025). Additional preclinical work should be conducted to study the possible utility of psychedelic medicine, including non‐hallucinogenic neuroplastogens, to treat dysfunctions associated with PAE.

## Author Contributions

Conceptualization: O.J.A. and T.G.E. Methodology: T.G.E. and O.J.A. Data curation: T.G.E. and T.D. Investigation: T.G.E. and T.D. Validation: T.G.E. and T.D. Formal analysis: T.G.E. Supervision: O.J.A. Funding acquisition: O.J.A. Visualization: T.G.E. and T.D. Project administration: O.J.A. Resources: O.J.A. Writing – original draft: T.G.E. and O.J.A. Writing – review and editing: T.G.E., O.J.A., and T.D.

## Funding

This work was supported by NIH Grants R01MH129282 (O.J.A.) and T32DA007268 (T.G.E.), as well as by the University of Michigan Eisenberg Family Depression Center Eisenberg Scholar Award (O.J.A.).

## Conflicts of Interest

The authors declare no conflicts of interest.

## Supporting information




**Figure 1—Supporting Information. Morphology of layer 5 pyramidal neurons**. Representative reconstructions of prefrontal cortex layer 5 neurons showing pyramidal morphology from control mice (left), PAE + saline treated mice (middle), or PAE + NBOH treated mice (right). Scale bar = 100 microns.

**Figure 2—Supporting Information. Passive membrane properties of PFC L5 pyramidal cells comparing PAE with saline treatment to control cells**. PAE does not alter the passive membrane properties of PFC L5 pyramidal neurons. Error bars represent SEM; ns, not significant. Full statistical details are provided in Table S1.

**Figure 3—Supporting Information. Passive membrane properties of PFC L5 pyramidal cells comparing neurons from PAE mice treated with either saline or NBOH**. NBOH treatment does not alter the passive membrane properties of PFC L5 pyramidal neurons in PAE. Error bars represent SEM; ns, not significant. Full statistical details are provided in Table S1.

**Figure 4—Supporting Information 1. Passive membrane properties of PFC L5 pyramidal cells comparing neurons from PAE mice with NBOH treatment to control neurons**. PAE followed by NBOH treatment does not alter the passive membrane properties of PFC L5 pyramidal neurons. Error bars represent SEM; ns, not significant. Full statistical details are provided in Table S1.

**Figure 4—Supporting Information 2. Effects of PAE followed by saline or NBOH on sEPSC frequency compared to a restricted subset of control neurons matching genotypes from the PAE + saline group. (A)** PAE + saline significantly decreased sEPSC frequency compared to control neurons**. (B)** PAE + NBOH had no significant changes in sEPSC frequency compared to control neurons. These results also held when using a Kruskal‐Wallis ANOVA test with corrections for multiple comparisons (Table S1).

**Table S1. Statistics**.

## Data Availability

Data needed to generate the figures will be made available upon reasonable request.
