# Supplementary figures and images for: A Single Dose of a Psychedelic Drug Repairs Prefrontal Cortex Synaptic Physiology in a Mouse Model of Prenatal Alcohol Exposure
Source: Brain Behav. 2026 Apr 20;16(4):e71406. doi: 10.1002/brb3.71406 (PMC13093898; doi:10.1002/brb3.71406)

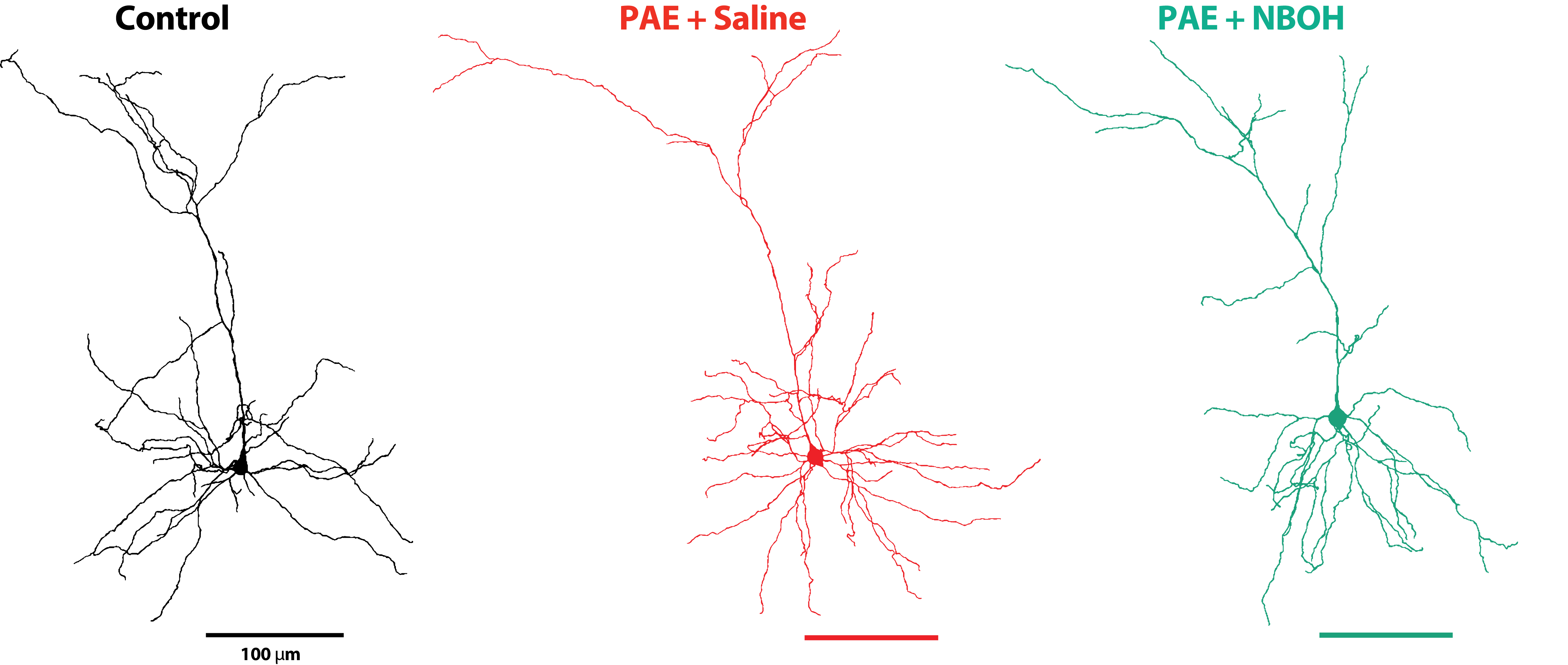

Supplement: Supplementary file 1 — Figure 1—Supporting Information. Morphology of layer 5 pyramidal neurons. Representative reconstructions of prefrontal cortex layer 5 neurons showing pyramidal morphology from control mice (left), PAE + saline treated mice (middle), or PAE + NBOH treated mice (right). Scale bar = 100 microns. [file BRB3-16-e71406-s004.png]

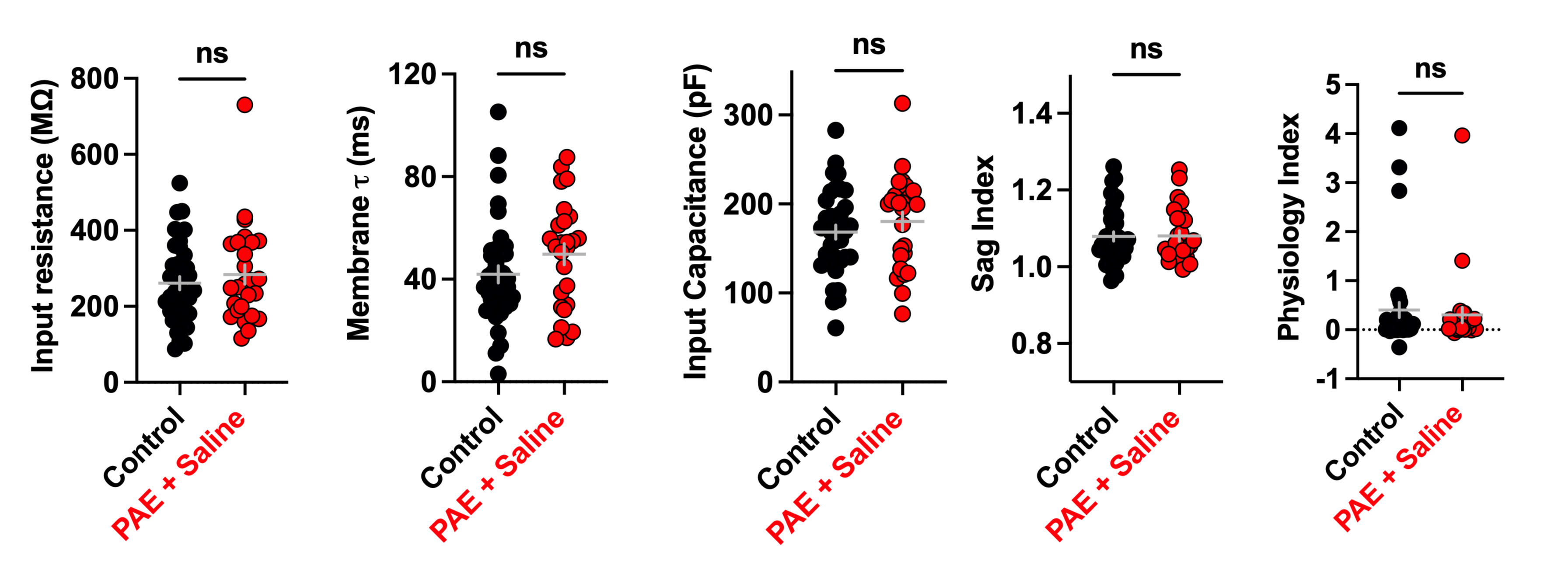

Supplement: Supplementary file 2 — Figure 2—Supporting Information. Passive membrane properties of PFC L5 pyramidal cells comparing PAE with saline treatment to control cells. PAE does not alter the passive membrane properties of PFC L5 pyramidal neurons. Error bars represent SEM; ns, not significant. Full statistical details are provided in Table S1. [file BRB3-16-e71406-s003.png]

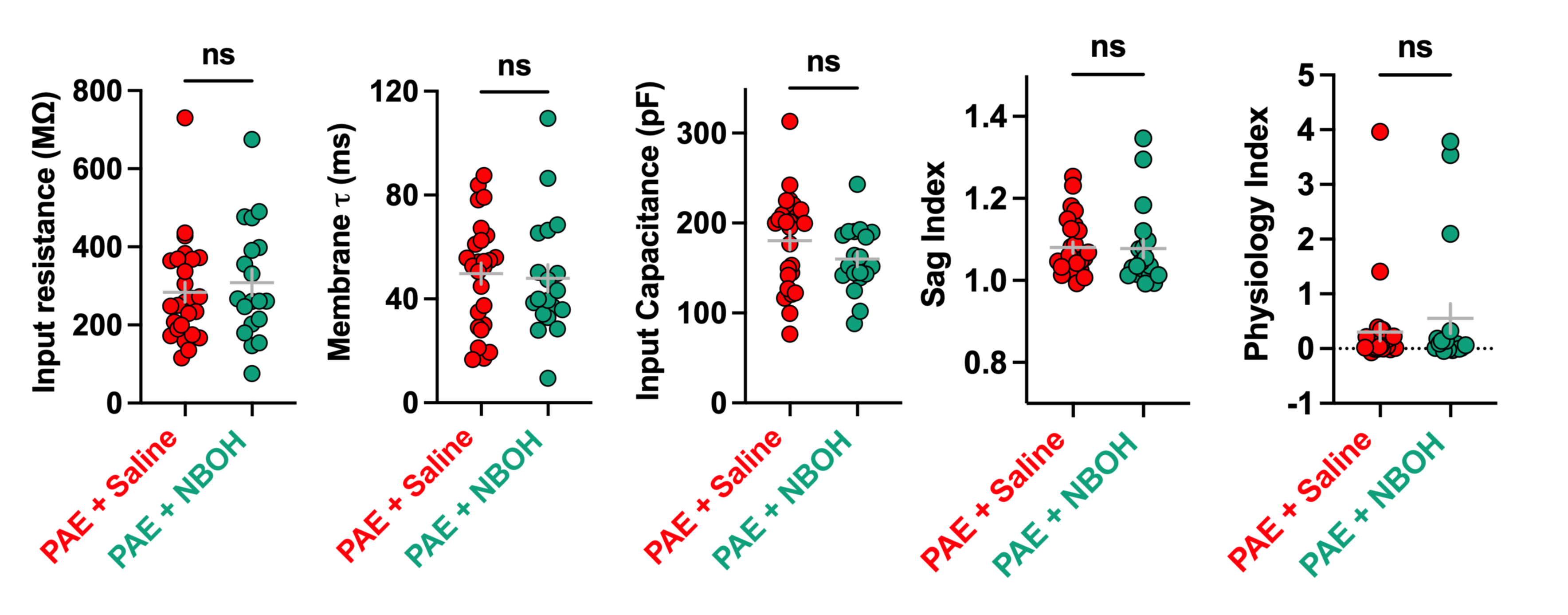

Supplement: Supplementary file 3 — Figure 3—Supporting Information. Passive membrane properties of PFC L5 pyramidal cells comparing neurons from PAE mice treated with either saline or NBOH. NBOH treatment does not alter the passive membrane properties of PFC L5 pyramidal neurons in PAE. Error bars represent SEM; ns, not significant. Full statistical details are provided in Table S1. [file BRB3-16-e71406-s006.png]

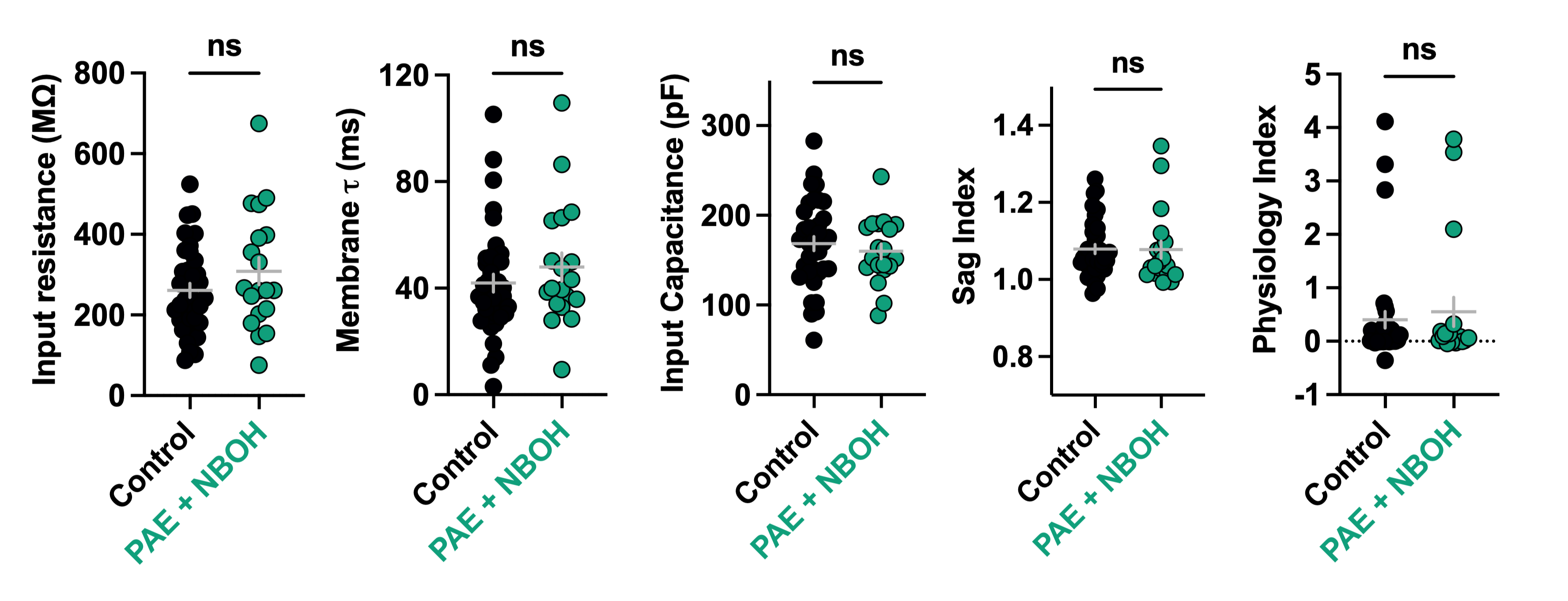

Supplement: Supplementary file 4 — Figure 4—Supporting Information 1. Passive membrane properties of PFC L5 pyramidal cells comparing neurons from PAE mice with NBOH treatment to control neurons. PAE followed by NBOH treatment does not alter the passive membrane properties of PFC L5 pyramidal neurons. Error bars represent SEM; ns, not significant. Full statistical details are provided in Table S1. [file BRB3-16-e71406-s001.png]

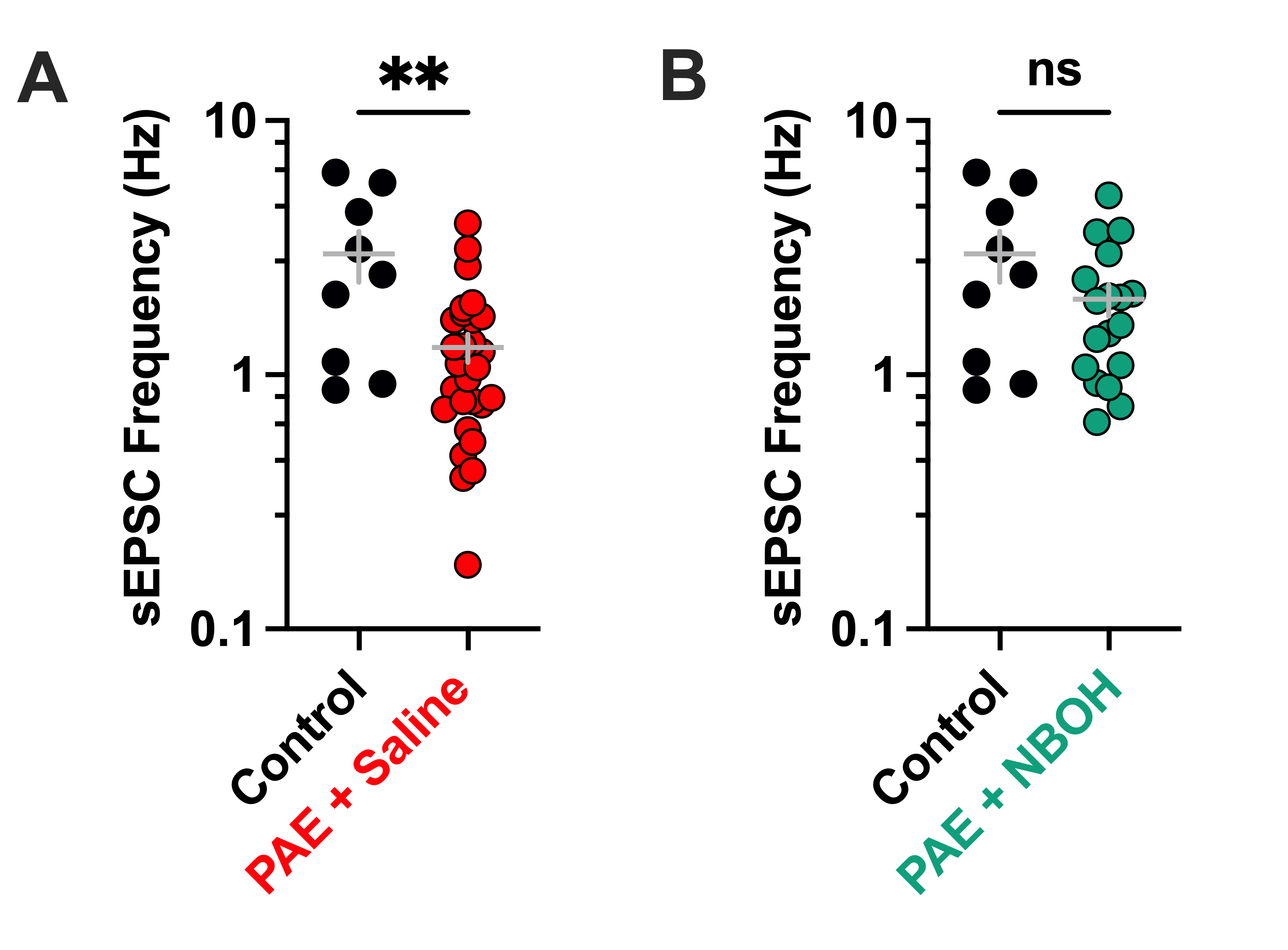

Supplement: Supplementary file 5 — Figure 4—Supporting Information 2. Effects of PAE followed by saline or NBOH on sEPSC frequency compared to a restricted subset of control neurons matching genotypes from the PAE + saline group. (A) PAE + saline significantly decreased sEPSC frequency compared to control neurons. (B) PAE + NBOH had no significant changes in sEPSC frequency compared to control neurons. These results also held when using a Kruskal‐Wallis ANOVA test with corrections for multiple comparisons (Table S1). [file BRB3-16-e71406-s005.png]
