# Supplementary material for: A Single Dose of a Psychedelic Drug Repairs Prefrontal Cortex Synaptic Physiology in a Mouse Model of Prenatal Alcohol Exposure
Source: Brain Behav. 2026 Apr 20;16(4):e71406. doi: 10.1002/brb3.71406 (PMC13093898; doi:10.1002/brb3.71406)
Supplement: Supplementary file 6 — Table S1. Statistics. [file BRB3-16-e71406-s002.pdf]

Table S1. Statistics.

Main Figure Statistics

Fig. 2

Maximum Firing Frequency (Max Firing Freq)

Group	n cells	Mean	SEM	Test	Statistic	Sum ranks	P-value
Control	37	32.00	1.386				
PAE + Saline	26	23.65	1.399				
Between groups				Two-tailed unpaired Mann-Whitney test	U=206	1459, 557	P=0.000071

Frequency-Current Gain (f-I Gain)

Group	n cells	Mean	SEM	Test	Statistic	Sum ranks	P-value
Control	37	.09216	.00522				
PAE + Saline	26	.06927	.00483				
Between groups				Two-tailed unpaired Mann-Whitney test	U=207	1458,558	P=0.000077

Total Spike Output

Group	n cells	Mean	SEM	Test	Statistic	df	P-value
Control	37	156.2	8.471				
PAE + Saline	26	123.5	9.84				
Between groups				Two-tailed unpaired t test	t=2.511	61	P=0.0147

Action Potential (AP) Max Rise Slope

Group	n cells	Mean	SEM	Test	Statistic	Sum ranks	P-value
Control	37	317.0	14.30				
PAE + Saline	26	234.6	11.86				
Between groups				Two-tailed unpaired t test	t=4.167	61	P=0.000099

AP Max Decay Slope

Group	n cells	Mean	SEM	Test	Statistic	df	P-value
Control	37	-63.72	2.452				
PAE + Saline	26	-43.64	1.305				
Between groups				Two-tailed unpaired t test	t=6.420	61	P=0.000000023

AP Half-width

Group	n cells	Mean	SEM	Test	Statistic	Sum ranks	P-value
Control	37	1.243	0.0439				
PAE + Saline	26	1.833	0.0565				
Between groups				Two-tailed unpaired Mann-Whitney test	U=58	761, 1255	P=0.00000000003

AP Amplitude

Group	n cells	Mean	SEM	Test	Statistic	df	P-value
Control	37	88.24	1.544				
PAE + Saline	26	89.23	1.611				
Between groups				Two-tailed unpaired t test	t=0.4338	61	P=0.6660

Spontaneous Excitatory Post-Synaptic Current (sEPSC) Frequency

Group	n cells	Mean	SEM	Test	Statistic	Sum ranks	P-value
Control	37	2.384	0.3351				
PAE + Saline	28	1.282	0.1617				
Between groups				Two-tailed unpaired Mann-Whitney test	U=346.5	1393, 752.5	P=0.0226

sEPSC Amplitude

Group	n cells	Mean	SEM	Test	Statistic	Sum ranks	P-value
Control	37	12.12	.3279				
PAE + Saline	28	11.43	.2837				
Between groups				Two-tailed unpaired Mann-Whitney test	U=414.5	706.5, 374.5	P=0.1725

sEPSC Decay Time Constant (Tau)

Group	n cells	Mean	SEM	Test	Statistic	Sum ranks	P-value
Control	37	5.111	0.1892				
PAE + Saline	28	6.648	0.3100				
Between groups				Two-tailed unpaired Mann-Whitney test	U=224	927, 1218	P=0.000058

Fig. 3

Maximum Firing Frequency (Max Firing Freq)

Group	n cells	Mean	SEM	Test	Statistic	Sum ranks	P-value
PAE + Saline	26	23.65	1.399				
PAE + NBOH	18	27.67	1.523				
Between groups				Two-tailed unpaired Mann-Whitney test	U=151.5	502.5, 487.5	P=0.0484

Frequency-Current Gain (f-I Gain)

Group	n cells	Mean	SEM	Test	Statistic	Sum ranks	P-value
PAE + Saline	26	.06927	.00483				
PAE + NBOH	18	.07406	.00334				
Between groups				Two-tailed unpaired Mann-Whitney test	U=175.5	526.5, 463.5	P=0.1657

Total Spike Output

Group	n cells	Mean	SEM	Test	Statistic	df	P-value
PAE + Saline	26	123.5	9.84				
PAE + NBOH	18	156.7	12.0				
Between groups				Two-tailed unpaired t test	t=2.145	42	P=0.0377

Action Potential (AP) Max Rise Slope

Group	n cells	Mean	SEM	Test	Statistic	df	P-value
PAE + Saline	26	234.6	11.86				
PAE + NBOH	19	246.4	11.08				
Between groups				Two-tailed unpaired t test	t=0.6993	43	P=0.4882

AP Max Decay Slope

Group	n cells	Mean	SEM	Test	Statistic	df	P-value
PAE + Saline	26	-43.64	1.305				
PAE + NBOH	19	-47.38	1.478				
Between groups				Two-tailed unpaired t test	t=1.888	43	P=0.0658

AP Half-width

Group	n cells	Mean	SEM	Test	Statistic	Sum ranks	P-value
PAE + Saline	26	1.833	0.0565				
PAE + NBOH	19	1.636	0.0488				
Between groups				Two-tailed unpaired Mann-Whitney test	U=141	704, 331	P=0.0143

AP Amplitude

Group	n cells	Mean	SEM	Test	Statistic	df	P-value
PAE + Saline	26	89.23	1.611				
PAE + NBOH	19	89.12	1.953				
Between groups				Two-tailed unpaired t test	t=0.04254	43	P=0.9663

Spontaneous Excitatory Post-Synaptic Current (sEPSC) Frequency

Group	n cells	Mean	SEM	Test	Statistic	Sum ranks	P-value
PAE + Saline	28	1.282	0.1617				
PAE + NBOH	18	1.981	0.2830				
Between groups				Two-tailed unpaired Mann-Whitney test	U=149.5	555.5, 525.5	P=0.0203

sEPSC Amplitude

Group	n cells	Mean	SEM	Test	Statistic	Sum ranks	P-value
PAE + Saline	28	11.43	.2837				
PAE + NBOH	18	11.07	.2326				
Between groups				Two-tailed unpaired Mann-Whitney test	U=203.	706.5, 374.5	P=0.2805

sEPSC Decay Time Constant (Tau)

Group	n cells	Mean	SEM	Test	Statistic	Sum ranks	P-value
PAE + Saline	28	6.648	0.3100				
PAE + NBOH	18	6.050	0.3900				
Between groups				Two-tailed unpaired Mann-Whitney test	U=182	728, 353	P=0.1185

Fig. 4

Maximum Firing Frequency (Max Firing Freq)

Group	n cells	Mean	SEM	Test	Statistic	Sum ranks	P-value
Control	37	32.00	1.386				
PAE + NBOH	18	27.67	1.523				
Between groups				Two-tailed unpaired Mann-Whitney test	U=230	1139, 401	P=0.0644

Frequency-Current Gain (f-I Gain)

Group	n cells	Mean	SEM	Test	Statistic	Sum ranks	P-value
Control	37	.09216	.00522				
PAE + NBOH	18	.07406	.00334				
Between groups				Two-tailed unpaired Mann-Whitney test	U=184	1185, 355	P=0.0067

Total Spike Output

Group	n cells	Mean	SEM	Test	Statistic	df	P-value
Control	37	156.2	8.471				
PAE + NBOH	18	156.7	12.0				
Between groups				Two-tailed unpaired Mann-Whitney test	t=0.0305	53	P=0.9758

Action Potential (AP) Max Rise Slope

Group	n cells	Mean	SEM	Test	Statistic	df	P-value
Control	37	317.0	14.30				
PAE + NBOH	19	246.4	11.08				
Between groups				Two-tailed unpaired t test	t=3.279	54	P=0.0018

AP Max Decay Slope

Group	n cells	Mean	SEM	Test	Statistic	Sum ranks	P-value
Control	37	-63.72	2.452				
PAE + NBOH	19	-47.38	1.478				
Between groups				Two-tailed unpaired t test	t=4.547	54	P=0.000031

AP Half-width

Group	n cells	Mean	SEM	Test	Statistic	Sum ranks	P-value
Control	37	1.243	0.0439				
PAE + NBOH	19	1.636	0.0488				
Between groups				Two-tailed unpaired Mann-Whitney test	U=85	788, 808	P=0.00000068

AP Amplitude

Group	n cells	Mean	SEM	Test	Statistic	df	P-value
Control	37	88.24	1.544				
PAE + NBOH	19	89.12	1.953				
Between groups				Two-tailed unpaired t test	t=0.3439	54	P=0.7323

Spontaneous Excitatory Post-Synaptic Current (sEPSC) Frequency

Group	n cells	Mean	SEM	Test	Statistic	Sum ranks	P-value
Control	37	2.384	0.3351				
PAE + NBOH	18	1.981	0.2830				
Between groups				Two-tailed unpaired Mann-Whitney test	U=330	1039, 501	P=0.9613

sEPSC Amplitude

Group	n cells	Mean	SEM	Test	Statistic	Sum ranks	P-value
Control	37	12.12	.3279				
PAE + NBOH	18	11.07	.2326				
Between groups				Two-tailed unpaired Mann-Whitney test	U=231	1138, 402	P=0.0677

sEPSC Decay Time Constant (Tau)

Group	n cells	Mean	SEM	Test	Statistic	Sum ranks	P-value
Control	37	5.111	0.1892				
PAE + NBOH	18	6.050	0.3900				
Between groups				Two-tailed unpaired Mann-Whitney test	U=243	946, 594	P=0.1089

sEPSC Frequency (One-way Kruskal-Wallis with corrections for multiple comparisons)

Group	n cells	Mean	SEM	Test	Statistic	Rank diff.	P-value (or adjusted P-value)
Control	37	2.384	0.3351				
PAE + Saline	28	1.282	0.1617				
PAE + NBOH	18	1.981	0.2830				
				Kruskal-Wallis ANOVA test	KW=6.978		P=0.0305
Control vs PAE + Saline				Multiple comparisons with corrections		14.50	P=0.0326
Control vs PAE + NBOH				Multiple comparisons with corrections		-0.812	P>0.9999

Maximum firing frequency (One-way ANOVA with corrections for multiple comparisons)

Group	n cells	Mean	SEM	Test	Statistic	Mean diff.	P-value (or adjusted P-value)
Control	37	32.00	1.386				
PAE + Saline	26	23.65	1.399				
PAE + NBOH	18	27.67	1.523				
				One-way ANOVA test	F=9.228		P=0.0003
Control vs PAE + Saline				Multiple comparisons with corrections		8.346	P=0.0001
Control vs PAE + NBOH				Multiple comparisons with corrections		4.333	P=0.0517

Total Spike Output (One-way ANOVA with corrections for multiple comparisons)

Group	n cells	Mean	SEM	Test	Statistic	Mean diff.	P-value (or adjusted P-value)
Control	37	156.2	8.471				
PAE + Saline	26	123.5	9.840				
PAE + NBOH	18	156.7	12.00				
				One-way ANOVA test	F=3.679		P=0.0297
Control vs PAE + Saline				Multiple comparisons with corrections		32.75	P=0.0272
Control vs PAE + NBOH				Multiple comparisons with corrections		-0.451	P=0.9994

Supplemental Figure Statistics

Fig. 2—Supporting Information

Input Resistance

Group	n cells	Mean	SEM	Test	Statistic	Sum ranks	P-value
Control	37	261.1	17.22				
PAE + Saline	26	283.7	25.40				
Between groups				Two-tailed unpaired Mann-Whitney test	U=447	1150, 886	P=0.6423

Membrane Time Constant (Tau)

Group	n cells	Mean	SEM	Test	Statistic	Sum ranks	P-value
Control	37	41.93	3.371				
PAE + Saline	26	49.76	4.081				
Between groups				Two-tailed unpaired Mann-Whitney test	U=357	1060, 956	P=0.0847

Input Capacitance

Group	n cells	Mean	SEM	Test	Statistic	df	P-value
Control	37	168.5	7.817				
PAE + Saline	26	180.5	10.26				
Between groups				Two-tailed unpaired t test	t=0.948	61	P=0.3469

Sag Index

Group	n cells	Mean	SEM	Test	Statistic	Sum ranks	P-value
Control	37	1.079	0.0120				
PAE + Saline	26	1.080	0.0137				
Between groups				Two-tailed unpaired Mann-Whitney test	U=480	1185, 831	P=0.9917

Physiology Index

Group	n cells	Mean	SEM	Test	Statistic	df	P-value
Control	37	0.4030	0.1547				
PAE + Saline	26	0.3011	0.1566				
Between groups				Two-tailed unpaired t test	t=0.4494	61	P=0.6548

Figure 3—Supporting Information

Input Resistance

Group	n cells	Mean	SEM	Test	Statistic	Sum ranks	P-value
PAE + Saline	26	283.7	25.40				
PAE + NBOH	19	308.6	33.95				
Between groups				Two-tailed unpaired Mann-Whitney test	U=219	570, 465	P=0.5312

Membrane Time Constant (Tau)

Group	n cells	Mean	SEM	Test	Statistic	Sum ranks	P-value
PAE + Saline	26	49.76	4.081				
PAE + NBOH	19	47.94	5.285				
Between groups				Two-tailed unpaired Mann-Whitney test	U=220	625, 410	P=0.5463

Input Capacitance

Group	n cells	Mean	SEM	Test	Statistic	df	P-value
PAE + Saline	26	180.5	10.26				
PAE + NBOH	19	159.8	8.279				
Between groups				Two-tailed unpaired t test	t=1.482	43	P=0.1456

Sag Index

Group	n cells	Mean	SEM	Test	Statistic	Sum ranks	P-value
PAE + Saline	26	1.080	0.0137				
PAE + NBOH	19	1.077	0.0225				
Between groups				Two-tailed unpaired Mann-Whitney test	U=211	634, 401	P=0.4185

Physiology Index

Group	n cells	Mean	SEM	Test	Statistic	df	P-value
PAE + Saline	26	0.3011	0.1566				
PAE + NBOH	19	0.5507	0.2740				
Between groups				Two-tailed unpaired t test	t=0.8406	43	P=0.4052

Figure 4—Supporting Information 1

Input Resistance

Group	n cells	Mean	SEM	Test	Statistic	Sum ranks	P-value
Control	37	261.1	17.22				
PAE + NBOH	19	308.6	33.95				
Between groups				Two-tailed unpaired Mann-Whitney test	U=296	999, 597	P=0.3444

Membrane Time Constant (Tau)

Group	n cells	Mean	SEM	Test	Statistic	Sum ranks	P-value
Control	37	41.93	3.371				
PAE + NBOH	19	47.94	5.285				
Between groups				Two-tailed unpaired Mann-Whitney test	U=298	1001, 595	P=0.3623

Input Capacitance

Group	n cells	Mean	SEM	Test	Statistic	df	P-value
Control	37	168.5	7.817				
PAE + NBOH	19	159.8	8.279				
Between groups				Two-tailed unpaired t test	t=0.6957	54	P=0.4896

Sag Index

Group	n cells	Mean	SEM	Test	Statistic	Sum ranks	P-value
Control	37	1.079	0.0120				
PAE + NBOH	19	1.077	0.0225				
Between groups				Two-tailed unpaired Mann-Whitney test	U=304.5	1102, 494.5	P=0.4219

Physiology Index

Group	n cells	Mean	SEM	Test	Statistic	df	P-value
Control	37	0.4030	0.1547				
PAE + NBOH	19	0.5507	0.2740				
Between groups				Two-tailed unpaired t test	t=0.5071	54	P=0.6142

Figure 4—Supporting Information 2

sEPSC Frequency

Group	n cells	Mean	SEM	Test	Statistic	Sum ranks	P-value
Control	9	2.991	0.6791				
PAE + Saline	28	1.282	0.1617				
Between groups				Two-tailed unpaired Mann-Whitney test	U=54	243, 460	P=0.0093

sEPSC Frequency

Group	n cells	Mean	SEM	Test	Statistic	Sum ranks	P-value
Control	9	2.991	0.6791				
PAE + NBOH	18	1.981	0.2830				
Between groups				Two-tailed unpaired Mann-Whitney test	U=57	150, 228	P=0.2317

sEPSC Frequency

Group	n cells	Mean	SEM	Test	Statistic	Rank diff.	P-value (or adjusted P-value)
Control	9	2.991	0.6791				
PAE + Saline	28	1.282	0.1617				
PAE + NBOH	18	1.981	0.2830				
				Kruskal-Wallis ANOVA test	KW=9.562		P=0.0084
Control vs PAE + Saline				Multiple comparisons with corrections		16.90	P=0.0118
Control vs PAE + NBOH				Multiple comparisons with corrections		6.306	P=0.6700
